# Circular RNAs, miRNAs, and Exosomes: Their Roles and Importance in Amyloid-Beta and Tau Pathologies in Alzheimer's Disease

**DOI:** 10.1155/np/9581369

**Published:** 2025-04-08

**Authors:** Mukaddes Pala, Senay Gorucu Yilmaz

**Affiliations:** ^1^Department of Physiology, Faculty of Medicine, Malatya Turgut Ozal University, Malatya, Türkiye; ^2^Department of Nutrition and Dietetics, Faculty of Health Science, Gaziantep University, Gaziantep, Türkiye

**Keywords:** Alzheimer's disease, circRNA, exosomes, miRNA

## Abstract

Alzheimer's disease (AD) is a devastating neurodegenerative disorder. The pathology of this disease is based on two basic mechanisms: amyloid-beta (A*β*) and tau fibrillation. Many genes and mechanisms have been identified as the primary causes of AD in clinical settings, and there have been exciting developments in drug treatments. Several molecules and biological structures regulate the genome outside of the standard DNA function. As in many diseases, circular RNAs (circRNAs), microRNAs (miRNAs), and exosomes (EXOs), investigated from different aspects of AD, are useful for treatment and diagnosis. This review examines two biological elements regarding their roles in the A*β*-tau pathology of AD and their potential as treatment targets. Importantly, the activities of miRNAs that play a role in these processes were evaluated.

**Trial Registration:** ClinicalTrials.gov identifiers: NCT04120493, NCT04969172, NCT04388982

## 1. Introduction

Alzheimer's disease (AD) is a progressive neurodegenerative disorder and the most common cause of dementia. It is characterized by the accumulation of amyloid-beta (A*β*) plaques and tau protein tangles in the brain, leading to neuronal cell death, brain atrophy, and cognitive decline. Symptoms typically include memory loss, confusion, difficulty with language, and behavioral changes. The exact cause of Alzheimer's is not fully understood, but it is believed to involve a combination of genetic, environmental, and lifestyle factors. The basic pathological features are as follows: A*β* plaques: abnormal accumulation of A*β* protein forms plaques in the brain. These plaques disrupt the communication between neurons and trigger an inflammatory response, leading to neuronal damage. A*β* is formed as a result of the enzymatic breakdown of amyloid precursor protein (APP). This process is impaired in Alzheimer's patients. Tau protein tangles: The tau protein is normally responsible for stabilizing microtubules inside neurons. In AD, tau protein becomes hyperphosphorylated and forms abnormal tangles (neurofibrillary tangles [NFTs]). These tangles disrupt the internal structure of neurons and prevent intracellular transport, causing loss of neuronal function and death. In AD, neurons are lost, especially in brain regions associated with memory and learning (hippocampus and cortex). The synaptic connections are weakened, leading to information processing and communication disorders. Brain cells, such as microglia and astrocytes, respond to inflammation caused by amyloid plaques and neuronal damage. However, chronic inflammation can further exacerbate neuronal damage. Oxidative stress is the accumulation of free radicals in cells and the inadequacy of antioxidant defense mechanisms. This impairs neuronal energy production and contributes to cell death [1].

## 2. AD Types and Variants

AD can be classified according to the age of onset and underlying causes: late-onset AD (sporadic Alzheimer's, LOAD) and early-onset AD (familial Alzheimer's, EOAD), Alzheimer's associated with mild cognitive impairment (MCI), Alzheimer's associated with Down syndrome. LOAD is the most common form and is usually observed in individuals over the age of 65. The exact cause is unknown, but genetic predisposition (e.g., APOE *ε*4 allele) and environmental factors (lifestyle, diet, cardiovascular risk factors) play a role. EOAD: a rare form that usually begins between the ages of 30–60. This form is associated with genetic mutations (e.g., mutations in *APP*, *PSEN1*, and *PSEN2*) and is inherited as an autosomal dominant trait. MCI is considered an early stage of AD. At this stage, individuals experience memory problems, but their daily activities are not yet severely affected. Some individuals with MCI may progress to AD over time. Individuals with Down syndrome have an additional copy of the *APP* gene. This increases the accumulation of A*β*, increasing the risk of developing Alzheimer's at an early age. Alzheimer's symptoms usually appear in individuals with Down syndrome at the age of 40–50.

AD is divided into two variants: posterior cortical atrophy (PCA) and logopenic primary progressive aphasia (LPA). PCA is characterized by deterioration in visual and spatial skills. Amyloid plaques and tau tangles are concentrated in the posterior parts of the brain. LPA: deterioration in language and speech skills is prominent. This variant is associated with the pathology of AD. In conclusion, AD is characterized by complex pathological processes such as A*β* plaques, tau tangles, neuronal loss, and inflammation. The disease is divided into different types according to the age of onset and underlying causes. Understanding these pathologies and types is critical for early diagnosis and development of targeted treatments.

### 2.1. A*β* Pathology

A*β* is a peptide derived from the APP, a transmembrane protein expressed in neurons. APP is cleaved by two enzymes, beta-secretase and gamma-secretase, in a process called amyloidogenic processing. This cleavage produces A*β* peptides, which can aggregate into oligomers, fibrils, and, eventually, insoluble plaques that accumulate in the brain.

• A*β* Plaques: These extracellular deposits are primarily composed of A*β*42, a highly aggregation-prone peptide. A*β* plaques disrupt neuronal communication, induce inflammation, and contribute to synaptic dysfunction and neuronal death.
• A*β* Oligomers: Soluble A*β* oligomers are considered more toxic than plaques. They interfere with synaptic function, impair long-term potentiation (LTP), and induce oxidative stress and mitochondrial dysfunction.

The “amyloid cascade hypothesis” posits that A*β* accumulation is the initial trigger in AD pathogenesis, leading to downstream effects such as tau pathology, neuroinflammation, and neurodegeneration.

### 2.2. Tau Pathology

Tau is a microtubule-associated protein that stabilizes microtubules in neurons and is essential for axonal transport and neuronal structures. In AD, tau becomes abnormally hyperphosphorylated, causing it to detach from microtubules and aggregate into paired helical filaments (PHFs) that form NFTs.

• NFTs: These intracellular aggregates disrupt microtubule stability, impair axonal transport, and lead to neuronal dysfunction and cell death. The spread of tau pathology is closely correlated with the progression of cognitive decline in AD.
• Tau Propagation: Pathological tau can spread transsynaptically from one neuron to another, propagating neurodegeneration throughout the brain.

Although A*β* pathology is thought to initiate the disease process, tau pathology is more closely associated with the severity and progression of cognitive symptoms.

Considering the importance of A*β* and tau in Alzheimer's research, they have attracted attention as diagnostic markers. Both A*β* and tau are critical biomarkers for the diagnosis of AD. A*β* levels can be detected in cerebrospinal fluid (CSF) or amyloid PET imaging, while tau pathology is assessed using tau PET imaging or CSF phospho-tau levels. They are central to studies of therapeutic targets. Most drug development studies for AD have focused on targeting A*β* and tau. Anti-A*β* therapies such as monoclonal antibodies (e.g., aducanumab and lecanemab) aim to clear A*β* plaques. Tau-targeted therapies such as tau aggregation inhibitors and tau immunotherapies are also being investigated. Understanding the interaction between A*β* and tau is crucial to understand AD pathogenesis. A*β* is thought to initiate the disease, whereas tau mediates neurodegeneration. Studies on these interactions may reveal new avenues for treatment. Identifying A*β* and tau pathology in preclinical stages (before the onset of symptoms) is a major focus of research, as early intervention may prevent or delay cognitive decline.

The known phenotypes of AD are classified as EOAD and LOAD, familial, and sporadic. However, behind the iceberg, researchers have reported the existence of subphenotypes based on their molecular characteristics and risk profiles. When the four classical types of AD are examined separately, there are data on the EV characteristics. However, no data comparing these four types have been reported. In a study investigating EVs in familial AD, EVs obtained from an iPSC cell line derived from a patient with the A246E mutation were injected into the hippocampus of mice (C57BL/6). Tau phosphorylation was detected in these EV-injected mice. This result suggests that EVs mediate tau phosphorylation in familial AD [2]. Ongoing research on EOAD and LOAD differences suggests that the frontal cortex has higher A*β* and NFTs in EOAD cases [3]. Exosomal research needs to be deepened in these two types, which are needed the most for AD diagnosis and determination of treatment targets. However, there is evidence for circular RNAs (circRNAs) for AD types [4]. A comprehensive study has demonstrated the existence of differential circRNA expression in vascular and Lewy body dementia. As a result, when characterizing exosomes (EXOs) in AD types, many criteria should be considered, and their circRNA contents should be detected and compared. Thus, it may be possible to identify the dark spots in the pathological spectrum of AD.

The entorhinal cortex and hippocampus are the brain regions affected by AD, and degenerative processes weaken neuronal communication between other brain regions associated with memory. The role of neural EXOs in brain biology is significant, as they enable communication between myelination, oligodendrocytes, and microglia [5]. In addition, glutamate and neurotransmitters are involved in triggering EXO release. For example, EXOs contained in oligodendroglia protect axons from oxidative stress through the delivery of two important molecules (catalase and superoxide dismutase-1) in the oxidative stress pathway [6]. Characterization of EVs from eight different brain regions showed that these molecules released from different cells of the CNS differ in number, size, morphology, protein, and RNA content [7]. The best data on vesicles in this study was obtained from the cerebellum. The smallest particles were observed in the thalamus. This study also highlights the differences in surface markers that allow vesicle identification. CD63 was less abundant than CD91 and CD9 in all the regions. These markers also vary among the different brain regions. The richness and diversity of these vesicles highlight the heterogeneity of the CNS. This diversity suggests that further studies are needed to determine the precise function of EVs in the CNS. When we consider this information in the context of exosomal circRNAs, confusion becomes clearer. There are compelling data on the composition of circRNAs in different brain regions. A meta-analysis using circMeta2 showed higher circRNA expression in the frontal cortex (12.274 circRNA cluster) than in the cerebellum (11.694 cluster) [8]. Another study analyzed 11,039 circRNAs from the brain and nonneuronal cells of 190 independent individuals, showing that 1526 circRNAs were enriched in dopamine and 3308 in pyramidal neurons. Of these, 12% were associated with AD-associated genes. The specific behavior of circRNAs may be a marker of synaptic specialization in AD [9]. Expanding these studies to all brain regions and characterizing EXO and circRNA contents can reveal the order in a normally functioning brain. Thus, it may be possible to understand the relationship between diseases and circRNA-targeted exosomal therapies can increase target specificity. In this context, we can describe the cooperation between EXOs and circRNAs as the “tailoring of the CNS system.”

### 2.3. CircRNAs and EXOs

#### 2.3.1. CircRNAs

CircRNAs are a class of noncoding RNAs with a covalently closed loop structure, which makes them highly stable and resistant to degradation by exonucleases. They are generated through a process called back-splicing, in which a downstream splice donor site is joined to an upstream splice acceptor site. In AD, circRNAs have been implicated in the regulation of key pathways associated with neurodegeneration. They are abundantly expressed in the brain and play critical roles in gene regulation by acting as microRNA (miRNA) sponges, modulating transcription, and interacting with RNA-binding proteins (RBPs).

• MiRNA Sponging: CircRNAs can act as competing endogenous RNAs (ceRNAs) by binding to miRNAs and preventing them from interacting with their target mRNAs.
• Protein Binding: Some circRNAs interact with RBPs, influencing their function or localization.
• Translation: Certain circRNAs can be translated into peptides or proteins, although this is uncommon.

CircRNAs are a diverse and functionally important class of RNAs. Their classification is based on origin, biogenesis, function, conservation, expression patterns, localization, and length. They play critical roles in gene regulation, cellular processes, and diseases, making them a focus of ongoing research in molecular biology and biomedicine.  Table 1 provides a concise overview of the diverse classifications of circRNAs based on their origin, biogenesis, function, conservation, expression patterns, localization, and length. Each type of circRNA has unique characteristics and roles in various cellular processes and diseases.

#### 2.3.2. EXOs

EXOs are small extracellular vesicles (30–150 nm) released by cells through the endosomal pathway that plays a key role in intercellular communication. EXOs are involved in several processes.

• Cell-to-Cell Signaling: EXOs transfer bioactive molecules between cells, influencing recipient cell behavior.
• Waste Management: They help cells remove unwanted or harmful substances.
• Disease Propagation: In neurodegenerative diseases, EXOs can spread pathological proteins, such as A*β* and tau.

EXOs are rising stars for drug delivery. Their characterization has shown that they carry important molecules involved in cellular communication and are biologically active. EXOs are a class of extracellular vesicles released from cells that carry molecular mail by packaging, vary according to cell type, and are released into the extracellular compartment by ectocytosis. They are part of the normal physiology of all cells, prokaryotes, and eukaryotes and have an average diameter of 100 nm [10]. The components vary depending on the cell from which they are born (mtDNA, dsDNA, ssDNA, viral DNA, lipids, mRNAs, miRNAs, circRNAs, pre-miRNAs, Y-RNAs, mtRNAs, tRNAs, tsRNAs, snRNAs, snoRNAs, piRNAs, cytokines, protein antigen presenters, tetraspanins, integrins, and cell adhesion molecules). Although their production purposes are not exactly known, when their contents, travel routes, and destinations are considered, it is not difficult to assume that they play a role in maintaining cellular homeostasis and removing unnecessary metabolites. What is important at this point is how EXOs find specific targets and how their decision-making processes operate. The answer to this question is hidden in the molecular structure and composition. EXOs are covered with a double-membrane structure, a double layer of phospholipids, and many molecules (heat shock proteins [HSPs], lipid rafts, immune regulatory molecules, cytoskeleton proteins, membrane fusion and transmembrane proteins, and marker and receptor proteins) [11]. EXOs originate endosomally via de novo formation of multivesicular bodies (MVBs) comprising early sorting endosomes (ESEs), late-sorting endosomes (LSEs), and intraluminal vesicles (ILVs) [10]. EXOs, which constitute heterogeneous populations, have different characteristics. When classified according to their heterogeneity, their sizes, cargo content, recipient cell characteristics, origins, common component proteins, and specific component proteins are important. Accordingly, the size heterogeneity was classified as ExoA (40–75 nm), ExoB (75–100 nm), or ExoC (100–160 nm). It stands out as a potential biomarker based on its content. These included Exo1 (CD63, nucleic acids, protein X), Exo2 (CD9, nucleic acids, protein Y), and Exo3 (CD81, nucleic acids, protein Z) in the tetraspanin group. The following functional EXOs were used: Exo *α* (transmission of survival signals to recipient cells), Exo *β* (transmission of proapoptotic signals to recipient cells), and Exo *γ* (immunomodulatory effect on recipient cells). Source-based EXOs: Exo I (brain-derived), Exo II (pancreatic-derived), and Exo III (liver-derived). Combinations of these molecules are responsible for EXO heterogeneity (e.g., Exo A, 1, *α*, I) (Table 2).

**Figure 1:**
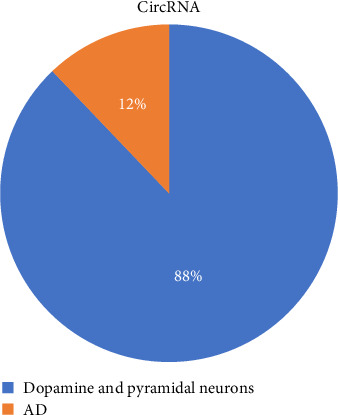
A cross-section of circular RNA (circRNA) distribution and its involvement in Alzheimer's disease (AD).

Biogenesis resembles the normal packaging processes [10]. In addition, EXOs include the GTPases, Rab, Syntenin-1, TSG101, ALIX, syndecan-1, ESCRT, ceramides, sphingomyelinases, and SNAREs. It is difficult to interpret the combined and separate effects of these molecules and to determine their functions because of molecular intersections during the transport of other vesicles (especially autophagy, lysosomal pathways, and Golgi-derived vesicles). Understanding the characteristic changes that they cause in recipient cells via cell culture systems may be helpful as they can reveal cell–cell communication through various mechanisms. These include localization to the target cell plasma membrane and intracellular signal activation via ligand-receptor binding, phagocytosis, micropinocytosis, receptor-mediated endocytosis, and direct membrane fusion with cytoplasmic transport to the cytoplasm [12]. During the cellular uptake of EXOs, cell membrane fusion with molecular mediators (v-SNARE, SNAREs, Rab GTPase, etc.) is essential. Surface proteins (tetraspanins and integrins) are effective for cellular recognition of EXOs. To some extent, the recognition of these molecules is limited. Thus, they can fuse with specific cells [13]. An understanding of the general characteristics of EXOs and their health and disease behaviors will help to understand their dynamics. Disruption of EXO dynamics can be directly or indirectly effective in the treatment of diseases. Changes that disrupt the integrity of EXOs can cause different results or unwanted combinations, and molecular contents can be transmitted. They induce cellular stress and damage under pathological conditions. Therefore, they are powerful molecules, and their instantaneous degradation is not the case. The ability of EXOs to reach long distances through the blood, lymph, and CSF can affect many systemic functions. Thus, they can be effective in treating diseases, such as cancer, AD, Parkinson's disease, and inflammatory processes. EXOs can cause disease on their own and have the potential to be biomarkers. Samples taken from the circulatory tract or specific tissues can aid in the diagnosis, prognosis, or detection of the disease before its onset. Thus, it may be possible to start early treatment for diseases or to prevent them. As stated previously, EXOs contain circRNA. Therefore, they are a class of covalently closed RNAs that do not have 5′ or 3′ ends [14]. Closure is a defense mechanism against exonuclease activity and prevents RNA degradation. Thus, its stability increases and stabilizes. This class of RNA functions as a protein and gene sponge. Their production is carried out via a cyclization mechanism with a lasso formed by back-splicing [15] and exon-skipping read-through. CircRNAs are classified into five categories: exonic, intronic, antisense, sense overlapping, and intergenic. CircRNAs can play a role in the regulation of gene expression, tumor mechanisms, cell proliferation, invasion, and pathology of many diseases by acting together with miRNAs as miRNA sponges. They are present in body fluids (blood, urine, saliva, semen, synovial fluid, and CSF), tissues, and EXOs. CircRNAs have various functions in EXOs, such as acting as miRNA sponges, inhibiting protein expression, regulating splicing, and regulating transcription and translation [15]. Their biogenesis begins in the nucleus and they are manufactured according to different types. They are then contained in EXOs. They exhibit unique properties. The number of tissue-specific circRNAs is high [16]. The data indicated the presence of more than 100 circRNAs in human serum. Their functions in neurodegenerative processes include EXO biogenesis and regulation of neuronal secretory vesicles [10]. In particular, some circRNAs are enriched in the brain and occur preferentially in neuronal cells through back-splicing. One study reported the expression of more than 11,039 circRNAs in dopamine and pyramidal neurons of 190 individuals [9]. Of these circRNAs, 12% were produced by AD-related genes (Figure 1).

These data suggest that circRNAs play a role in regulating synaptic function. Although circRNAs alone are effective in the pathology of diseases, circRNAs in structures with high cargo potential, such as EXOs, are important because of their stability and abundance. One of the best-known functions of circRNAs is as miRNA sponges. For example, Cdr1as, which is expressed in the mammalian brain, contains more than 70 miR-7 binding sites and is the exact target of mature miR-671 [17]. The diversity versus specificity arises from the fact that EXOs reflect the type and state of the cells from which they originate. However, EXOs of different origins may have the same content. We believe that EXOs can respond to general and special-purpose metabolic reactions. These changing units are likely RNA subsets that are specific to specific functions. The analyses showed that the circRNA types of EXOs differed according to tissue origin. This difference shows that circRNAs are not randomly incorporated into EXOs but rather are actively, consciously, and sequentially incorporated [18]. However, cargo selection in EXOs remains unclear. Finally, the effect of miRNAs on the EXO-circRNA-miRNA triad has been studied for a long time, and the dynamics of these epigenetic molecules are well known. However, the functions of circRNA-mediated miRNAs involved in this combination remain unclear. The localization of miRNAs on circRNAs, their association–dissociation dynamics, and their packaging and release in EXOs involve complex processes.

### 2.4. MiRNAs

MiRNAs are epigenetic mediators that are ~21–23 nt in length in the small, single-stranded, noncoding RNA class. Its functions include transcriptional repression and protein degradation at the gene level. They also have a wide range of target specificities owing to their short sequence characteristics. An miRNA can be specific to more than one target, or more than one miRNA can use the same target. These dynamic molecules are unlikely to be found in EXOs. Studies have shown that EXO-miRNAs play a role in important mechanisms such as cell migration, apoptosis, proliferation, and autophagy [19]. MiRNA biogenesis is well known. The two main sources of miRNAs direct the formation of mature miRNAs from pre-miRNAs. Many miRNAs are encoded by genes transcribed by RNA polymerase II and are processed by various mechanisms [20]. There are four possible mechanisms by which miRNAs are located in EXOs: sphingomyelinase 2, hnRNP, miRISC, and sequence-dependent pathways involving the 3′-UTR of miRNAs. EXO-miRNAs play a role in the posttranscriptional regulation of their target cells and are more stable than free miRNAs. Because miRNAs packaged into EXOs are not exposed to enzymes in biological fluids, they escape RNase processing. This stability may result in permanent disease effects in diseases [21]. Altered EXO-miRNA profiles are known to predict CNS diseases prior to their occurrence. EXO-miRNA EXOs in the adult brain determine the fate of NSCs and maintain the neurogenic niche [22]. The cellular transfer of EXO-miRNAs is a novel mechanism of genetic exchange. EXO-miRNAs not only have different expression profiles than cellular and free miRNAs but also have different expression profiles in daughter cells than in parental cells [23].

### 2.5. EXOs, MiRNAs, and CircRNAs in the AD Brain

AD is a brain-based disease. To use the brain as a source and target for the diagnosis and treatment of the disease, the blood–brain barrier (BBB) must be crossed. There are various mechanisms through which these three molecules cross the BBB. These mechanisms facilitate access to information that enables their effective use as diagnostic and therapeutic tools [24].

**Figure 2:**
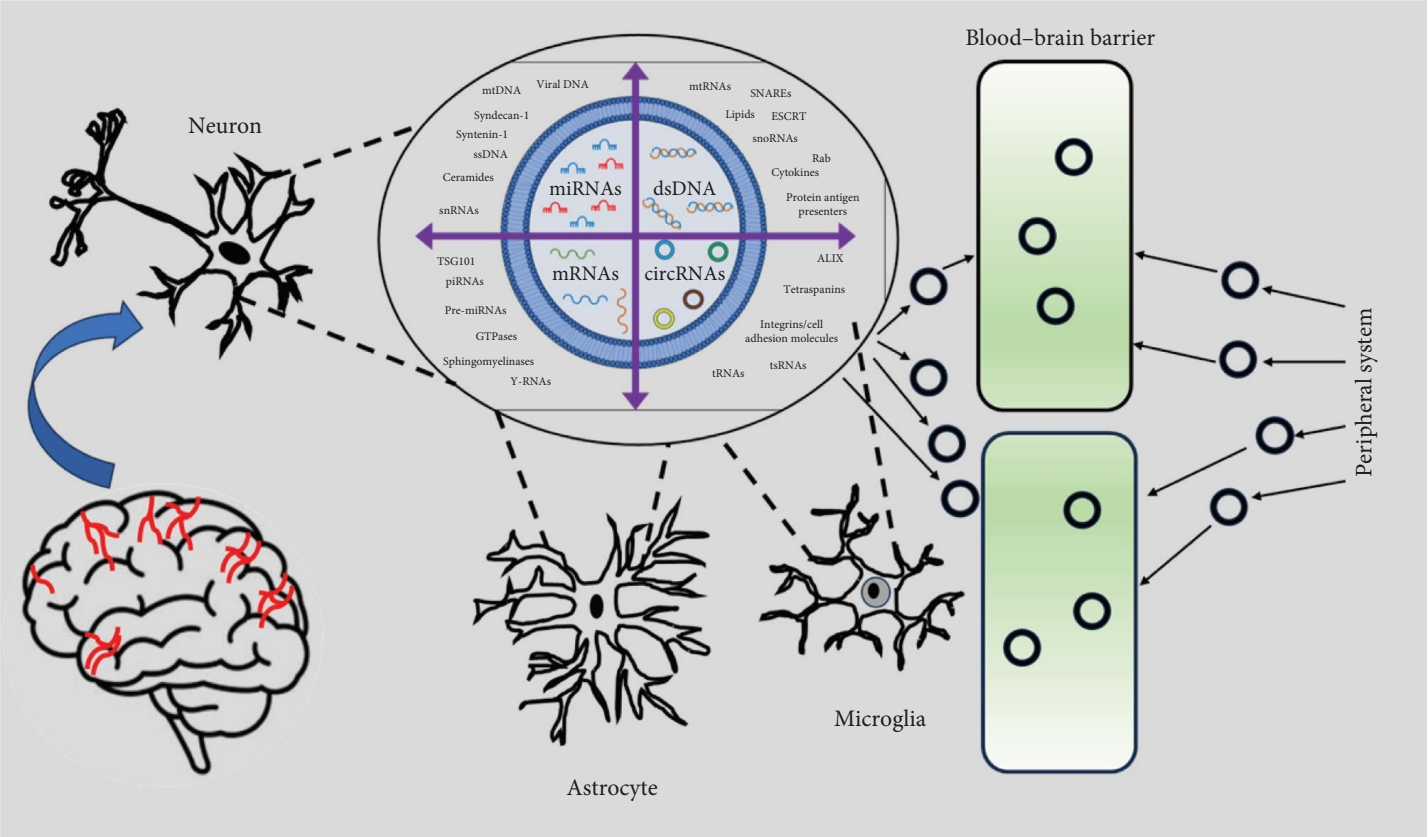
Efflux of exosome-circular RNA (circRNA)–microRNA (miRNA) across the blood–brain barrier (BBB).

The BBB is a highly selective barrier that protects the brain from harmful substances in the bloodstream while allowing essential nutrients to pass through. However, certain molecules and structures, such as circRNAs, miRNAs, and EXOs, have been shown to cross the BBB under specific conditions. Here, we provide an overview of how these entities interact with the BBB via circRNAs, miRNAs, and EXOs. CircRNAs are a type of noncoding RNA that play regulatory roles in gene expression and cellular processes. CircRNAs do not typically cross the BBB directly. However, they can also be packaged into extracellular vesicles (e.g., EXOs) and transported across the BBB. EXOs containing circRNAs can be secreted by cells, enter the bloodstream, and potentially cross the BBB through transcytosis or other vesicle-mediated transport mechanisms. MiRNAs are small noncoding RNAs that post-transcriptionally regulate gene expression. MiRNAs can cross the BBB either freely or within extracellular vesicles, such as EXOs. Some studies have suggested that miRNAs can passively diffuse across the BBB, especially if the barrier is compromised (e.g., in disease states). MiRNAs are often encapsulated in EXOs that can cross the BBB via receptor-mediated transcytosis or other vesicular transport mechanisms. MiRNA transport across the BBB is important for intercellular communication and may play a role in neurological diseases. EXOs are small extracellular vesicles (30–150 nm) that carry proteins, lipids, and nucleic acids (e.g., circRNAs, miRNAs) for intercellular communication. EXOs are known to efficiently cross the BBB. EXOs can cross the BBB via transcytosis, a process by which vesicles are transported through endothelial cells. EXOs may interact with specific receptors on the BBB endothelial cells, facilitating their uptake and transport. In conditions such as inflammation, cancer, or neurodegenerative diseases, the BBB may become more permeable, enhancing EXO transport. EXOs have been explored as potential drug delivery vehicles to target the brain because of their natural ability to cross the BBB. The ability of circRNAs, miRNAs, and EXOs to cross the BBB can be unified into three key points. 1- Direct and Indirect Transport: CircRNAs and miRNAs generally rely on EXOs or other vesicles to cross the BBB rather than passing independently. 2- BBB Permeability: The ability of these molecules to cross the BBB may be affected by the health of the barrier (e.g., compromised in disease states). 3- Therapeutic Potential: Understanding how circRNAs, miRNAs, and EXOs cross the BBB is critical for developing treatments for brain tumors, AD, and neurological disorders such as stroke. In this process, EXOs play a role in many pathologies in AD through the circRNAs and miRNAs they contain (Figure 2).

EXOs have been shown that EXOs contribute to AD pathogenesis by managing the flow of data between neurons and glia and affecting A*β* production and accumulation via their miRNA content [25, 26]. Several mechanisms trigger A*β* and tau fibrillation in AD. We know the separate effects of EXOs (according to their content), circRNAs, and miRNAs on AD. CircRNAs regulate miRNA sponge activity due to the presence of miRNA binding sites. Since this process occurs in EXOs, it can be a solid sign of AD since the process is precise and permanent. In addition, using EXO packages as a treatment target is a reliable way to transport cargo protected from cellular degradation processes. Peptides involved in AD processes were discovered in EXOs via the cleavage of APP sequences [27]. These vesicles contain APP, terminal components, and proteases, indicating the important role of EXOs in AD pathology. EXOs are ejected as a result of *β* and *β* secretases breaking down APP in early endosomes and delivering A*β* to MVBs. EXOs ensure that A*β* lives in its normal process, while the failure of A*β* to be excreted in large numbers in EXOs causes excessive A*β* transmission in neuronal communication. In the human brain, hyperphosphorylated tau accumulates in toxic forms in critical regions of learning and memory. It is thought that the filaments that spread in AD are Tau proteins released by EXOs [28]. The BBB is the most important barrier in the human brain. The easy penetration of EXOs through the BBB allows effective signaling. It also allows cargo to be loaded onto EXOs to reach the brain. There are different hypotheses regarding this transport pathway: transcytosis, efflux, and carrier-mediated [29]. In this transport pathway, where surface proteins of EXOs play an important role, CD63 levels are high, especially in EXOs expressed in brain endothelial cells, and CNS penetration is effective [30]. In a study that bioinformatically examined exosomal circRNA–miRNA expression in plasma samples of patients with AD, specific circRNAs and miRNAs were identified, and their mRNA targets were also suggested [31]. Gui et al. [32] demonstrated that miRNA expression profiles are altered in CSF EXOs. A study in which AD-specific 16-miRNA was selected based on apolipoprotein *ɛ*4 (APOE *ɛ*4) allele status showed that exosomal miRNA may be a suitable peripheral screening tool for AD [33]. Beta-site APP-cleaving enzyme 1 is a *β*-secretase required for A*β* production. Various miRNAs affect the expression of beta-secretase 1 (BACE-1) [34]. *γ*-Secretase, which is responsible for A*β* formation, consists of four components: presenilin-1, nicastrin, pen-2, and aph [35]. Nexin 27 (Snx27) inhibits *γ*-secretase activity, and, therefore, A*β* production by binding to presenilin [36].

The activities of UBE2D3, UBE2B, and UBCH10, which play a role in the clearance and ubiquitination of A*β*, are regulated by miRNAs [37–40]. Neprilysin, a protease that cleaves A*β*, belongs to the M13 family of metallopeptidases [41]. Moreover, neprilysin mRNA and protein levels and enzymatic activity are reduced in patients with AD [42]. Another group of proteases that degrades A*β* is matrix metalloproteinases (MMPs) [43].

Cathepsin D is another enzyme that degrades A*β* [44]. Overexpression of miRNA, which downregulates cathepsin D, impairs the degradation of A*β* [45]. According to these results, miRNAs were present at all stages of A*β* formation. Therefore, miRNAs involved in A*β* formation may be potential targets in AD.

Glycogen synthase kinase-3 (GSK-3), which phosphorylates tau, is upregulated in AD patients [46]. Nimodipine, a calcium channel blocker, suppresses tau phosphorylation by reducing GSK-3 mRNA levels [47]. In addition, miRNAs involved in the activity of phosphatase and tensin homolog (PTEN)/phosphatidylinositol-3 kinase (PI3K)/AKT and extracellular signal-regulated protein kinase (ERK1/2) signaling pathways cause GSK-3 and tau phosphorylation [48, 49]. Another kinase that phosphorylates tau is cyclin-dependent kinase-5 (cdk5) [50, 51]. In the 3xTg-AD mouse knockout model, miRNAs induce tau phosphorylation through the activation of Fyn and serine/threonine-protein kinase 2 (SRPK2) [52, 53]. MiRNAs involved in the regulation of tau phosphorylation may serve as therapeutic targets for AD.

A mouse study demonstrated the GM1-A*β* relationship by revealing that GM1 is located in the central region of A*β* plaques [54]. In another mammal, the monkey, this pair was discovered during the early stages of AD [55]. In contrast, disruption of neuronal endocytosis, including Rab5, which is involved in the regulation of endosomal expansion and early endocytosis, accelerates GM1-related EXO release and increases amyloid fibrillation. As a result, impaired APP metabolism causes A*β* accumulation, and when it reaches a limit that lysosomal or glial cells cannot cope with, the toxic protein is released from the cell and spreads to the brain with EXOs.

EXOs are likely to mediate the progression of tau pathology. An EXO-mediated mechanism has been suggested in the M1C neuroblastoma tautopathy model. EXO-tau has also been detected in CSF samples [56]. In transgenic mice with a tau model, EXOs were found to contain tau, and it was observed that they initiated pathology depending on the threshold values [57].

Neuronal bridges lost during the normal aging process match the age, gender, and various comorbidities of patients with AD. EXOs experience a golden age with their applications in the aging process. Developments in dermatology, particularly as an antiaging agent, are drawing attention. Studies have suggested that EXOs facilitate synaptic transmission by facilitating neuron-oligodendrocyte connectivity. The mechanism required is that oligodendrocytes trigger EXO secretion with essential myelin components, which results in EXOs being accepted by neurons and cellular survival [58]. However, the variability of EXOs under physiological conditions and difficulty in maintaining their activation are among the major problems. MiRNAs have been widely studied in EXO-related studies, The best example is miR-193b. This miRNA targets the 3′-UTR of APP mRNA and, strikingly, lower exosomal concentrations were detected in AD patients than in controls. This result is associated with increased A*β* formation [59]. One of the most important processes in AD is neuroinflammation, which can be modulated by EXOs. In one example study, EXOs obtained from age-matched AD patients and healthy individuals were found to activate the complement pathway and lead to disruption of plasma membrane integrity and neurotoxicity [60]. Some data suggest that A*β* and tau protein accumulation occurs throughout the CNS via EXOs [61]. These confusing findings regarding the benefits and harms of EXOs in AD trigger discussions about their potential as a treatment source. In this regard, individuals over the expected age of onset of 50 could be sampled from both sexes at certain intervals. An EXO association study conducted by repeating the same individuals and randomly including individuals (if alive) in this sample can help explain their activities. Of course, there are biasing factors. Since healthy aging of these individuals cannot be guaranteed, comorbidities will also be included in the process. In addition to the varying parameters, it can be explained whether EXO behaviors are the cause or result of AD. The role of EXOs and phagocytic microglia in the spread of pathological proteins in AD should be evaluated. In addition, it should be noted that transcripts contained in EXOs may trigger the synthesis of these proteins. Identifying EXOs and their cargo during the physiological aging process may shed light on this complexity.

It is questionable whether EXO exhibits gender differences. Studies have indicated that there are some differences. In a study of different age groups, plasma samples collected twice 5 years apart showed that EVs decreased with age [62]. Studies show that sex differences in EXOs differ between men and women depending on tissues, biological sources, and diseases [63]. Therefore, this is an expected result for AD patients. An important study on this subject involves brain EXOs. Sex and EV levels were investigated in aging mice (C57BL/6 J), and different EV and EV levels were determined at different stages. Female mice expressed more plasma membrane-derived EXOs with age, whereas no difference was observed in male mice. The situation was different in the mitochondria. Microvesicles increased with age in both sexes. These and similar data suggest that aging processes in the female brain are associated with susceptibility to AD [64].

Whether AD is the cause or result of a single clinical condition remains under debate. Comorbidities that trigger this disease may be important in this process. Numerous studies have been conducted on this topic. The most common diseases include depression, cardiovascular disease, osteoporosis, diabetes, and obesity, and are associated with the onset of AD [65]. When these AD components are considered in terms of EXOs, confusion grows because each side factor contributes to AD through its exosomal mechanisms. When age and sex are added to these diseases, it may be difficult to identify EXOs, miRNAs, and circRNAs and to associate them with AD. CircRNAs may help explain the contribution of comorbidities in AD by preserving their selectivity owing to their miRNA specificity and special circular structures. It may be limited in revealing the effects of diseases associated with AD, especially those with age and sex bias. A notable study on this subject showed the role of circRNAs in postoperative neurocognitive disorders [66]. Thus, the circRNA–miRNA–mRNA connection is of great importance. Regarding circRNA and exome-related mechanisms in AD, research can clarify comorbidities. EXOs are rich in circRNAs [67]. In terms of comorbidities in AD, the detection of EXO circRNAs in different combinations, especially in AD, may help explain age, gender, and comorbidities and determine treatment targets.

## 3. The Role of CircRNAs, MiRNAs, and EXOs in AD Pathogenesis

Despite significant advances in our understanding of AD, the underlying molecular mechanisms remain elusive. Recent studies have highlighted the involvement of circRNAs and EXOs in AD pathogenesis, offering new insights into disease progression and potential therapeutic targets. This review summarizes the current understanding of how circRNAs and EXOs contribute to AD, with a focus on their roles in A*β* and tau pathology, intercellular communication, and neuroinflammation.

### 3.1. CircRNAs: Regulators of Gene Expression in AD

CircRNAs are a class of noncoding RNAs with a covalently closed loop structure, which makes them highly stable and resistant to degradation. They are abundantly expressed in the brain and play critical roles in gene regulation by acting as miRNA sponges, modulating transcription, and interacting with RBPs. In AD, circRNAs have been implicated in the regulation of key pathways associated with neurodegeneration.

#### 3.1.1. CircRNAs and A*β* Pathology

A*β* plaques, which are formed by the aggregation of A*β* peptides, are a hallmark of AD. CircRNAs influence A*β* production and clearance by regulating the expression of enzymes involved in APP processing, such as *β*-secretase. For example, circRNAs can sponge miRNAs that target BACE1 mRNA, thereby indirectly increasing A*β* production. Additionally, circRNAs are involved in dysfunction of the ubiquitin-proteasome system and autophagy, both of which are critical for A*β* clearance.

#### 3.1.2. CircRNAs and Tau Pathology

Hyperphosphorylation of the tau protein leads to the formation of NFTs, another hallmark of AD. CircRNAs can modulate tau phosphorylation by regulating the activity of kinases (e.g., GSK-3*β*) and phosphatases (e.g., PP2A). For instance, circRNA_0001445 has been reported to sponge miR-23b, which targets the tau kinase GSK-3*β*, thereby influencing tau phosphorylation levels [68].

### 3.2. MiRNAs and A*β* Pathology

1. A*β* Production: MiRNAs regulate the expression of genes involved in the amyloidogenic pathway, such as APP and BACE1. Example:
  • miR-16: Downregulates APP expression and reduces A*β* production [69].
  • miR-107: Regulates BACE1 expression, and its downregulation in early AD leads to increased BACE1 activity and A*β* accumulation.
2. A*β* Clearance: miRNAs also influence the expression of proteins involved in A*β* clearance, such as neprilysin and insulin-degrading enzyme (IDE). Example: miR-34a inhibits neprilysin, reducing A*β* clearance [70].

### 3.3. MiRNAs and Tau Pathology

1. Tau Phosphorylation: MiRNAs regulate kinases and phosphatases involved in tau phosphorylation, a key feature of AD. miR-132: Downregulated in AD, leading to increased tau phosphorylation via the activation of kinases, such as GSK-3*β* [71]. miR-26b promotes tau hyperphosphorylation by targeting protein phosphatase 2A (PP2A) [72].
2. Tau Aggregation: Some miRNAs influence tau aggregation and microtubule stability. miR-219 is involved in maintaining tau homeostasis and is downregulated in AD.

### 3.4. MiRNAs and Neuroinflammation

1. Microglial Activation: MiRNAs regulate microglial activation, which contributes to neuroinflammation in AD. For example, miR-155 promotes proinflammatory microglial activation and is upregulated in AD. miR-124 suppresses microglial activation and is downregulated in AD.
2. Cytokine Production: MiRNAs modulate inflammatory cytokine expression. Example: miR-146a is upregulated in AD and promotes inflammation by targeting complement factor H (CFH), an anti-inflammatory protein [73].

### 3.5. MiRNAs and Synaptic Dysfunction

1. Synaptic Plasticity: miRNAs regulate genes involved in synaptic plasticity and neuronal communication. For example, miR-132 is critical for synaptic function and neuronal health, and its downregulation in AD contributes to synaptic loss [74]. miR-134 regulates synaptic plasticity and is dysregulated in AD.
2. Synaptic Protein Expression: miRNAs influence the expression of synaptic proteins such as PSD-95 and synaptophysin. Example: miR-125b targets PSD-95, leading to synaptic dysfunction.

### 3.6. MiRNAs and Neuronal Apoptosis

1. Cell Survival Pathways: MiRNAs regulate pathways involved in neuronal survival and apoptosis. For example, the miR-29 family is downregulated in AD, leading to increased neuronal apoptosis. miR-34a promotes neuronal death by targeting antiapoptotic genes, such as BCL2 [75].

### 3.7. MiRNAs as Biomarkers in AD

1. Early Diagnosis: Dysregulated miRNAs in the blood, CSF, or EXOs can serve as noninvasive biomarkers for early AD detection. Example: miR-125b, miR-135a, and miR-29b are dysregulated in AD patients' blood and CSF [76].
2. Disease Progression: Specific miRNA profiles correlate with AD severity and progression. For example, miR-34a levels increase with disease progression.

### 3.8. Therapeutic Potential of miRNAs in AD

1. miRNA Mimics: Restoring downregulated miRNAs (e.g., miR-132 and miR-107) can reduce A*β* production, tau pathology, and synaptic dysfunction [77].
2. miRNA Inhibitors: Blocking overexpressed miRNAs (e.g., miR-155 and miR-146a) can reduce neuroinflammation and neuronal death [78].
3. Delivery Systems: EXOs or nanoparticles can be used to deliver therapeutic miRNAs to the brain, targeting specific AD pathways [79].

The regulation profiles of key miRNAs with extensive function in AD are summarized in Table 3.

The potential of miRNAs in AD remains unclear. However, there are also challenges associated with their use. The first is specificity, which ensures that miRNA-based therapies target only the intended pathways without off-target effects. The second is the development of effective and safe delivery systems (e.g., EXOs and nanoparticles) to transport miRNAs across the BBB. Finally, biomarker validation was performed. Validation of miRNA biomarkers in larger patient groups for clinical use. MiRNAs play a central role in the pathogenesis of AD by regulating fundamental processes, such as A*β* production, tau pathology, neuroinflammation, synaptic dysfunction, and neuronal death. Their dysregulation makes them valuable biomarkers for early diagnosis and promising therapeutic targets. Advances in miRNA-based therapies and delivery systems have great potential for treating AD and for improving patient outcomes [80].

### 3.9. EXOs: Mediators of Intercellular Communication in AD

EXOs are small extracellular vesicles (30–150 nm) secreted by cells that play a crucial role in intercellular communication. They carry a variety of cargoes, including proteins, lipids, and nucleic acids (e.g., miRNAs and circRNAs), and can cross the BBB. In AD, EXOs have been implicated in both the propagation of pathological proteins and the modulation of neuroinflammation.

#### 3.9.1. EXOs and A*β* Pathology

EXOs facilitate the spread of A*β* peptides between neurons and glial cells. They have been shown to carry A*β* and APP, contributing to the extracellular deposition of A*β* plaques. Moreover, EXOs can influence A*β* clearance by transporting enzymes involved in A*β* degradation, such as neprilysin and IDE.

#### 3.9.2. EXOs and Tau Pathology

EXOs are also involved in the intercellular transmission of pathological tau. They can package and transport hyperphosphorylated tau seeds, thereby promoting the spread of tau pathology across brain regions. This process is believed to contribute to the progressive nature of AD. Additionally, EXOs can carry circRNAs that regulate tau phosphorylation, further linking exosomal cargo with tau pathology.

### 3.10. The Interplay Between CircRNAs and EXOs in AD

The interaction between circRNAs and EXOs represents a novel axis in AD pathogenesis. EXOs can serve as vehicles for the transport of circRNAs between cells, enabling the regulation of gene expression in the recipient cells. For example:

• CircRNA Transfer via EXOs: EXOs released by neurons or glial cells can carry circRNAs that modulate A*β* and tau pathways in recipient cells. This transfer can amplify pathological processes such as increasing A*β* production or promoting tau hyperphosphorylation.
• Regulation of Neuroinflammation: Both circRNAs and EXOs are involved in regulating neuroinflammation, a key driver of AD progression. Exosomal circRNAs can modulate the activation of microglia and astrocytes, influencing the release of proinflammatory cytokines and exacerbating neuronal damage.

## 4. Therapeutic Implications

Understanding the roles of circRNAs and EXOs in AD opens new avenues for therapeutic intervention. Potential strategies include the following.

1. Targeting CircRNAs: Silencing or overexpressing specific circRNAs can modulate A*β* and tau pathology. For example, inhibition of circRNAs that promote BACE1 expression or tau phosphorylation could reduce A*β* production and tau aggregation.
2. Modulating Exosomal Cargo: Engineering EXOs to carry therapeutic circRNAs or other molecules could provide a targeted approach to deliver treatments across the BBB. EXOs can also be used as biomarkers for early AD diagnosis, given their role in disease progression.
3. Combination Therapies: Combining circRNA-based therapies with EXO modulation can enhance therapeutic efficacy by simultaneously addressing multiple pathological pathways.

CircRNAs, miRNAs, and EXOs have emerged as key players in the molecular landscape of AD. Their roles in regulating A*β* and tau pathology, as well as their interplay in intercellular communication, highlight their potential as therapeutic targets. Further research into the mechanisms by which circRNAs and EXOs contribute to AD pathogenesis is critical for developing novel diagnostic tools and treatments for this devastating disease. By unraveling the complex interactions between these molecules, we may move closer to effective strategies to prevent or halt the progression of AD.

### 4.1. EXO, CircRNA, and MiRNA-Targeted Therapies in AD

MiRNA-based therapy is an attractive option for treating neurodegenerative diseases and will soon become the next generation of therapeutic drugs [81]. MacroRNAs cannot pass through the BBB because they are more than 400 Daltons, in size and are not fat soluble. Therefore, they are not suitable for the treatment of brain injuries. Owing to their small size and inherent properties, miRNAs can be effective against brain damage. According to the clinicaltrials.gov database, miRNAs have been evaluated in phase IV trials [82]. In addition, the low toxicity of miRNA-based therapies and the use of EXOs to deliver miRNAs to different body parts make the design and production of miRNA-based drugs advantageous [83]. Additionally, very few miRNAs have entered the clinical stage and can be brought to market in a short time.

CircRNAs are good therapeutic targets owing to their high stability. However, nanoparticle or EXO delivery systems for cellular targeting are limited. In addition, the plasmid or RNAi strategies used in loss-of-function studies have limited application potential. However, a good understanding of the behavior of exomes that supports cellular communication and perhaps treatment in normal and AD pathology can provide exomes for therapeutic purposes. The potential contents of EXOs in healthy tissues and cells can be used as a source for other damaged tissues and cells. A known example of this is the use of cord blood cells in therapy. In particular, the EXO content in embryonic tissue and the developmental process can be a solution not only in AD but also in many diseases [84]. The identification of EXOs via the regeneration of tissues and cells and their potential use in the maturation process could aid in the treatment of A*β* and tau pathology in AD. Stem cells have great potential in the treatment of many diseases. Stem cell-derived EXOs are advantageous for treatment because of their immunity, nontoxicity, easy availability, lack of preservation, and nontumorigenic properties [85]. What is the status of stem cell-based EXO therapy for AD? EXOs can treat a damaged BBB by supporting neurogenesis and angiogenesis through their anti-apoptotic, anti-inflammatory, and antioxidant effects, thus providing a solution for many diseases in addition to AD [86].

In one study, the potential of human neural stem cell-derived EXOs to protect neuroblastoma cells was investigated. The effects of neural stem cell EXOs in reducing the amount of A*β* and tau were detected [87]. This potential of EXOs is also valid for mesenchymal stem cell-derived EXOs. One problem is a low numerical load. EXOs, which are driven by hypoxic conditions and HSP activation, exhibit increased anti-inflammatory and antioxidant properties [88]. EXOs are known to play messenger roles in AD processes and contain APP and tau products [89]. In AD, the EXO content of damaged neurons not only contains APP and tau but also transmits them to other neurons. The yin–yang effect of EXOs can be used to reverse AD processes. The miRNAs and circRNAs present in EXOs have great potential in this regard. With the miRNA and circRNA contents of natural, healthy EXOs, natural EXOs can be targeted by individuals.

EXO–circRNA–miRNA-based therapies are particularly prevalent in the field of cancer and regenerative medicine. They demonstrated the regenerative effect of mouse embryonic fibroblast-derived EXOs on sciatic nerve injury in an experimental mouse sciatic nerve injury model [90]. Another study demonstrated the regenerative effect of MEFs on diabetic wound healing [91]. The most extensive studies on this subject have involved miRNAs. The siRNA drug Patisiran, which received FDA approval in 2018, was used to treat polyneuropathy caused by hereditary transthyretin-mediated (hATTR) amyloidosis [92]. Clinical studies have aimed to suppress or activate miRNAs by mimicking them. However, more effective and specific treatments are possible with subjective circRNA sponges that can make miRNAs more specific. Using this duo as an exosomal cargo can provide superiority in passing through the BBB and also protect enzymatic processes. Clinical trial records state that AMT-130, designed for Huntington's disease, is a phase II study that involves adeno vector-mediated treatment. In another phase II study, the liposomal mimic of MIRX34, a miR-34a mimic, was used in advanced solid tumors [93]. Although EXO-based therapies are new, potential tools remain to be developed. Some clinical studies involve EXO therapy, whereas others involve its use as a drug delivery vehicle. Human biological samples and plant EXOs are preferred EXO sources. However, compliance with good manufacturing practices (GMPs) is required for human therapies. A phase II randomized, double-blind, placebo-controlled study to evaluate the safety and efficacy of CD24. Overexpressed EXOs to prevent clinical deterioration in patients with moderate or severe COVID-19 infection. A phase I study of EXOs as drug cargo is examines the exosomal delivery of curcumin to treat colon tumors. This study aimed to solve the bioavailability problems of orally administered curcumin. Clinical studies on circRNAs have focused on circRNA vaccines [94]. The biggest handicap in this system is its degradation and low utilization during arrival at the target. The advantages of their delivery with encapsulation vehicles containing nanoparticles and EVs are being investigated. Clinical studies on the EXO–circRNA–miRNA axis have limited phase data when each is considered separately. The changing dynamics of these molecules in tissues, cells, and physiological aspects make it difficult to identify specific molecules. Detailed studies are required for all three molecules. The fact that there has been no definitive attempt yet to determine their presence in AD shows the urgency of this issue.

However, there remain challenges to overcome when using this technology. Off-target gene silencing is a major problem in miRNA and circRNA research. CRISPR systems and circRNA–miRNA sponges are currently being tested to reduce off-target effects. The fact that circRNAs and miRNAs target multiple tissues and mRNAs is a serious problem. This problem can only be solved by identifying specific circRNAs and miRNAs they will carry. Nanoparticle applications are feasible in preclinical studies, but their size-dependent toxicity must be adjusted. Additionally, transcriptional activators or repressors can be used to increase or suppress the expression of miRNAs and circRNAs. Because the vectors used in circRNA technology target intronic sequences, incorrectly spliced linear and circRNAs may result in nonspecific products and may be pathogenic. In this case, in vitro synthesis of circRNA molecules may solve this problem. Synthetic miRNAs and circRNAs can trigger the immune system. Chemical modifications of these molecules and coating them with various RBPs can reduce their immunogenicity [95].

There have been phase I/II studies based on EXOs in AD. One of them is the safety of EXOs derived from allogeneic adipose mesenchymal stem cells in patients with mild-to-moderate dementia. Although there are no circRNA studies, biomarker studies for miRNAs are in the majority.

## 5. Conclusion

Many miRNAs contribute to the pathophysiology of AD by regulating intracellular signaling pathways. Since miRNAs regulate the activity of target mRNAs via base pairing and suppress protein synthesis, they can be used for the identification of biomarkers and therapeutic targets. Thus, miRNAs may provide new treatment options for patients with AD. Using miRNAs as biomarkers for AD may lead to early detection of the disease, leading to better clinical outcomes resulting from early intervention. Additionally, the identification of new biomarkers will lead to the discovery of applicable treatment strategies for AD and improve the prognosis of patients. Collectively, these features support the regulatory roles of circRNAs at the transcriptional and posttranscriptional levels and offer potential as biomarkers or adjuvant therapies for AD. The earlier stabilization of these molecules in mRNA and their presentation with easy techniques such as real-time PCR may enable their use as primary tools in diagnosis and treatment. However, more advanced techniques are required to treat these diseases. Currently, circRNA treatment is available. These include CRISPR-Cas-based gene therapies, immunotherapies, protein replacement, and phytochemicals. Additionally, treatments can be developed using EXO–miRNA–circRNA complexes derived from healthy tissues and cells. Similar to experimental mouse embryonic fibroblast-derived EXOs, which have shown therapeutic success in mouse disease models, their applicability in humans can also be tested.

In addition, discoveries are being made based on gene regulatory mechanisms, such as SeekRNA, and new doors are being opened for both existing therapeutic problems and the treatment of unsolved diseases.

### 5.1. Future Perspectives

When developing EXOs, circRNA, and miRNA-based therapeutic approaches, their stability, tissue or cell specificity, and size should be carefully evaluated. Therefore, it may be advantageous to use all three molecules together to develop an effective tool. However, subsequent studies should examine in detail the use of nanoparticles, encapsulation, and CRISPR systems for these molecules. When the latest technologies are examined, siRNAs appear to be the most advantageous. CircRNA activity can be regulated using circRNA-specific siRNA. In the case of AD, it may be possible to regulate the transcription of genes that play an active role in the production of A*β* and tau. It may be possible to deliver specific circRNA–miRNA sponges to the target using exosomal bombardment. This method can sometimes be achieved by circulation and sometimes by a nasal spray, as has been tried before.

## Figures and Tables

**Table 1:** Summarizing the classification of circular RNAs (circRNAs) based on various criteria.

Classification criteria	Type of circRNA	Description
Origin	Exonic circRNAs (ecircRNAs)	Derived from exons of protein-coding genes; often function as miRNA sponges.
	Intronic circRNAs (ciRNAs)	Derived entirely from introns; often localized in the nucleus.
	Exon–intron circRNAs (EIciRNAs)	Contain both exonic and intronic sequences; regulate transcription.
	Intergenic circRNAs	Originate from intergenic regions (between genes).

Biogenesis	Lariat-driven circularization	Formed via exon skipping and lariat formation.
	Intron-pairing-driven circularization	Complementary sequences in flanking introns facilitate back-splicing.
	RBP-driven circularization	RNA-binding proteins (RBPs) promote circularization by bridging splice sites.

Function	miRNA sponges	Sequester miRNAs to regulate gene expression.
	Protein sponges or decoys	Interact with proteins to modulate their activity.
	Protein translation templates	Contain IRES elements and can be translated into peptides or proteins.
	Transcriptional regulators	Regulate transcription by interacting with RNA polymerase II or other factors.
	Scaffolds for protein complexes	Serve as platforms for assembling multiprotein complexes.

Conservation	Conserved circRNAs	Found across multiple species, indicating functional importance.
	Species-specific circRNAs	Unique to specific organisms, potentially contributing to species-specific traits.

Expression patterns	Tissue-specific circRNAs	Expressed in specific tissues or cell types.
	Developmentally regulated circRNAs	Expression changes during development or differentiation.
	Disease-associated circRNAs	Dysregulated in diseases like cancer, neurodegeneration, and cardiovascular diseases.

Localization	Nuclear circRNAs	Primarily, ciRNAs and EIciRNAs regulate transcription in the nucleus.
	Cytoplasmic circRNAs	Mostly, exonic circRNAs function as miRNA sponges or are involved in translation.

Length	Short circRNAs	Typically, less than 500 nucleotides.
	Long circRNAs	Can be several kilobases in length, often containing multiple exons.

**Table 2:** Classification of exosomes (based on their different characteristics).

Size heterogeneity-based (nm)	Content-based	Function-based	Source-based	Common component protein-based	Specific component protein-based (types and conditions of the parent cells of the exosome)
ExoA (40–75)	Exo1 (CD63, nucleic acids, protein X)	Exo *α* (transmission of survival signals to recipient cells)	Exo I (brain-derived)	Membrane fusion and transport-related proteins (Ras-associated binding [Rab]-GTPases, annexin, and heat shock proteins [HSPs] such as HSP60, HSP70, and HSP90)	cGMP-dependent protein kinase 1 (PKG1)

ExoB (75–100)	Exo2 (CD9, nucleic acids, protein Y)	Exo *β* (transmission of proapoptotic signals to recipient cells)	Exo II (pancreatic-derived)	MVB-related proteins (tumor ALG-2-interacting protein X [ALIX], a susceptibility gene 101 [TSG101], and vacuolar protein sorting-associated protein 4 [VPS4])	X-box-binding protein 1 (NFX1)

ExoC (100–160)	Exo3 (CD81, nucleic acids, protein Z)	Exo *γ* (immunomodulatory effect on recipient cells)	Exo III (liver-derived)	Transmembrane cross-linked proteins (intercellular adhesion molecule 1 [ICAM-1], tetraspanin-8 [TSPAN8], CD106, CD82, CD81, CD63, CD53, CD37, and CD9)	Epidermal growth factor receptor (EGFR)

	Tetraspanin	—	—	Other proteins (integrins, actin, myosin, cofilin, tubulin)	—

**Table 3:** Key miRNAs in AD and their functions.

MiRNA	Function in AD	Expression in AD
miR-132	Regulates synaptic plasticity, tau phosphorylation, and neuronal health	Downregulated
miR-107	Regulates BACE1 expression and A*β* production	Downregulated
miR-29 family	Regulates APP expression and neuronal apoptosis	Downregulated
miR-34a	Promotes neuronal apoptosis and inhibits A*β* clearance	Upregulated
miR-155	Promotes neuroinflammation and microglial activation	Upregulated
miR-146a	Enhances neuroinflammation by targeting anti-inflammatory proteins	Upregulated
miR-124	Suppresses microglial activation and inflammation	Downregulated
miR-125b	Targets synaptic proteins and promotes tau phosphorylation	Upregulated

## Data Availability

Data sharing is not applicable to this article as no datasets were generated or analyzed during the current study.
